# Odor of Fatty Acids: Differences in Threshold and Perception Intensity Between Sexes

**DOI:** 10.3390/foods14162777

**Published:** 2025-08-10

**Authors:** Daniela Diana, Giorgia Sollai

**Affiliations:** Department of Biomedical Sciences, University of Cagliari, 09042 Monserrato, CA, Italy; d.diana6@studenti.unica.it

**Keywords:** palmitic acid, oleic acid, linoleic acid, gas chromatography–olfactometry (GC-O) technique, olfactory function, Sniffin’ Sticks, females and males, smell, orthonasal pathway

## Abstract

In humans, food choice, nutrient intake, and meal size are strongly influenced by sense of smell. It is known that individuals differ in their olfactory abilities and may show a normal, reduced, or absent sense of smell. Previous findings have also suggested that males are more deficient than females in their olfactory performance. Recent studies have shown that humans could perceive the odor of free fatty acids, providing information about the nutritional content of foods. The aim of this research was to study the orthonasal perception of palmitic, oleic, and linoleic fatty acids in 70 healthy subjects (38 females and 32 males). First, participants were classified as normosmic or hyposmic by means of the Sniffin’ Sticks test. Second, the ability to detect the odor of fatty acids was assessed using the gas chromatography–olfactometry technique, a combination of sensory and instrumental analysis that allows for simultaneous chromatographic separation and odor evaluation by a human subject. Finally, the olfactory threshold to fatty acids was also evaluated by means of a three-way forced-choice test, using a presentation procedure of ascending concentration with seven dilution steps. The results highlighted differences in perception ability, perception intensity, and olfactory threshold in relation to the lipophilicity of the molecule, olfactory function, and sex. Our findings confirm the human ability to perceive the odor of fatty acids, with females and normosmic individuals performing better than males and hyposmic ones, respectively. They also show that the intensity of perception increases with the decreasing lipophilicity of fatty acids; consequently, the olfactory perception threshold also decreases.

## 1. Introduction

Fatty acids are common in foods and occur mainly in the ester form of a triglyceride, also known as a triacylglyceride; the three fatty acids of a triglyceride, attached to a glycerol molecule, can all be the same or two or three different ones [1,2]. In addition to triglycerides, various foods also contain low concentrations of free fatty acids [2,3]. Fatty acids of dietary interest, such as linoleic, oleic, and palmitic acids, are specifically described as long-chain, 18-carbon carboxylic acids, either unsaturated (linoleic and oleic) or saturated (palmitic). Fatty acids perform various functions within biological systems, contributing in energetic (available reserve of calories that can be mobilized when needed from adipose tissue), structural (formation of myelin, composition of the plasma membrane), and functional terms (synthesis and secretion of adipokines, thermogenesis, and synthesis of sexual hormones, adrenal hormones, vitamin D, and bile acids). In addition, polyunsaturated fats act as precursors of biologically active molecules involved in the regulation of inflammatory processes, blood pressure, platelet aggregation, coagulation, and renal function [4,5,6,7,8,9,10]. Dietary fats also contribute to the absorption of fat-soluble vitamins and carotenoids [11]. The excessive consumption of fats, particularly widespread in the Western world, represents a health risk: although not directly responsible, fats contribute to the onset of overweight, obesity, diabetes, cardiovascular diseases, and tumors [12,13,14,15,16,17,18].

Olfaction plays a significant role in food selection and meal size determination, leading to long-term implications, influencing body weight, energy balance, and the maintenance and promotion of human well-being, and contributing to both nutritional aspects and social relationships [19,20,21,22,23]. Most people who experience olfactory disorders report that food is less tasty and less pleasant and that these conditions change their eating and cooking habits [24,25]. People with a low sense of smell (hyposmia) tend to prefer highly palatable, salty, and spicy foods, rich in simple sugars or fats, over fruits, vegetables, and acidic and bitter foods [25,26,27,28,29]. These foods, often highly processed and high in calories, contribute significantly to the taste of food and influence the reward system in the brain [30,31,32]. A diet based on the abundant consumption of high-calorie-density foods, especially when associated with a sedentary lifestyle, is a major cause of obesity [33], defined as an epidemic in 1997 by the WHO, and of metabolic diseases [34]. In particular, highly processed foods, especially those with added sugar, play a key role in the development of non-communicable diseases [35]. Furthermore, the possible presence of olfactory dysfunction can lead to this caloric food and weight gain; therefore, a high-fat diet can worsen the olfactory deficit due to the pro-inflammatory state it causes [36,37,38,39].

Females are commonly accepted to have a better olfactory performance than males; however, some studies report that there are no sex-related differences in the olfactory function between individuals, leaving the topic still up for debate [40,41,42,43]. Some studies involving large samples of participants reported that olfactory detection or identification abilities are similar between females and males, especially when considering normosmic individuals [40,43,44]. Previous studies from our laboratories have shown that females perceive a higher number of individual molecules extracted from a complex mixture and also with higher intensity, explaining why they also perceive the complex odor (from which the molecules were extracted) with higher intensity [42]. Females show better olfactory function than males even in conditions of obesity or overweight, both in adults and the elderly [23,41]. Factors considered potentially responsible for the generation of sex-related differences in olfactory abilities are neuroendocrine (e.g., fluctuations associated with estrogen levels or the menstrual cycle) [45,46], social (males appear to be less interested in olfactory stimuli and are less familiar with odors) [47,48], cognitive (females perform better than males in episodic olfactory memory) [49,50], and genetic (the functionality and expression of Kv1.3 channels influence the olfactory function of both sexes, making females better performers) [51,52].

Despite the importance of fats and fatty acids in human nutrition and food choices, the ability to perceive their odor remains a topic of debate [53]. Some studies suggest that humans, through their sense of smell, can both detect the fat (and therefore caloric) content of foods and distinguish between different long-chain fatty acids in the gaseous phase [2,30]. Other studies, on the contrary, suggest that the perceived odor of fatty acids is actually that of their oxidation products, and that their perception and discrimination within foods may occur mainly via the retronasal route [2,30,54,55,56]. Furthermore, inter-individual variability in the detection threshold has also been suggested: the higher the concentrations of free fatty acids in the stimuli, the lower the subjective fat detection threshold [11].

Based on these considerations, the main objective of this study was to evaluate the ability of individuals to perceive the odor of palmitic (saturated), oleic (mono-unsaturated), and linoleic (polyunsaturated) fatty acids, as eluted from the chromatographic column. Using gas chromatography–olfactometry, a combination of sensory and instrumental analyses that allows for chromatographic separation and simultaneous odor evaluation by a human subject, we were able to establish with certainty that the odorant molecule perceived by the participant was indeed that of the fatty acid, and also assess its intensity. Once we established that fatty acids were smelled via an orthonasal pathway, we also assessed the corresponding olfactory threshold. Since we did not find previous studies that considered differences related to the sex and olfactory function of individuals, the second objective was to investigate the olfactory threshold and perception intensity of fatty acids in relation to the sex (males vs. females) and olfactory function (normosmia vs. hyposmia) of the participants.

## 2. Materials and Methods

### 2.1. Subjects

The search for volunteers who wanted to join the study panel was carried out through public announcements at the University of Cagliari. A total of 70 healthy subjects (38 females and 32 males; age 27.1 ± 1.1 years; BMI 18.5–24.99 Kg/m^2^), non-smokers and with a COVID-19 infection having occurred at least 12 months before, were selected. During the recruitment phase, it was necessary to explain to the candidates the aim of the study, the required duration, and the experimental procedure. The volunteers were selected on the basis of information collected through questionnaires or interviews to ensure that none of them had evident olfactory dysfunctions or were suffering from a cold or any other disorder that could interfere with their perceptive abilities. For all participants, the exclusion criteria were the presence of metabolic, inflammatory/autoimmune, neurodegenerative, cognitive, tumoral, respiratory, or cardiovascular diseases [37,57,58,59,60,61,62,63,64,65,66,67,68].

All volunteers were strictly prohibited from eating, drinking (except water), chewing gum, and/or smoking for 2 h before the experiment. Each volunteer was required to arrive in the laboratory at least 15 min before the start of the measurements to read the experimental protocol approved by the local ethics committee (Prot. PG/2021/14278, 22 September 2021) and sign the informed consent.

### 2.2. Evaluation of Olfactory Sensitivity

The orthonasal olfactory function of each subject was assessed by means of the “Sniffin’ Sticks Extended Test” (SSET); (Burghart Instruments, Wedel, Germany), widely used for olfactory screening and also internationally recognized for its validity in the health field [69]. The SSET is based on sniffing pens containing a felt soaked in an odor and includes three subtests: Threshold (Thre-test), Discrimination (Dis-test), and Identification (Id-test) test. The olfactory threshold (score 0–16) is assigned using a scale of increasing concentrations of the same odor, with a forced choice mechanism. To conduct the experiments, 16 triplets of pens are available (16 increasing concentrations). Each triplet consists of two pens containing a solvent, while the third (target pen, which the participant must identify) is filled with the test odor (n-butanol). The starting triplet (first reversal) is the one in which the participant identifies the target pen for the first time twice in a row. Subsequently, the triplet decreases until the participant makes an error, at which point it increases again (second reversal), and so on for seven reversals. The olfactory threshold is defined as the average of the dilution steps of the last 4 reversals. Olfactory discrimination (score 0–16) is assigned using 16 triplets of pens: each consists of two pens containing the same odor and a third filled with a different odor (target pen, which must be identified by the participant). During the identification test (score 0–16), consisting of 16 pens containing everyday odors familiar to the participants, they must choose between four possibilities that are presented to them. During the test, the experimenter fills out a protocol in which the scores obtained for each sub-test are reported. The sum of the scores obtained with the Thre-test, Dis-test, and Id-test allows to the total TDI to be obtained, through which the subjects are classified for their olfactory performance. In our experiments, the reference values used for classifying subjects as normosmic, hyposmic, or functionally anosmic took into account sex and age according to previous studies. Specifically, the lower limits for normosmia were those corresponding to the 10th percentile, separately for sex and age group, as reported by Hummel et al. [70].

### 2.3. Mass Spectrometry–Gas Chromatography–Olfactometry (MS-GC-O) Technique

The experiments were conducted using a gas chromatograph (Agilent 6890N; Santa Clara, CA, USA) simultaneously coupled to an olfactometer (Gerstel ODP3; Mülheim an der Ruhr, Germany) and a mass spectrometer (Agilent model 5973; Santa Clara, CA, USA) [71,72]. The carrier gas was ultrapure helium at a constant flow rate of 1.2 mL/min. Specifically, 1 µL of a fatty acid mixture composed of 20 mg/mL of palmitic acid (PA), oleic acid (OA), and linoleic acid (LA) was injected into the GC, and at the outlet of the GC column, the flow was split in a 1:1 ratio between the olfactometer and the mass spectrometer. The volatile compounds were compared with the mass spectrum present in the NIST2014 library (US National Institute of Standards and Technology; Gaithersburg, MD, USA), in order to confirm their purity and the absence of oxidation products.

The chromatographic column used was an HP-INNOWax (30 m × 0.25 mm × 0.50 µm) (Agilent 19091N-233; Agilent technologies, Santa Clara, CA, USA). The injector temperature was 250 °C, while the MS interface temperature was 260 °C. The oven temperature was set as 40 °C (0.2 min), 40 °C/min up to 100 °C (2 min), 10 °C/min up to 200 °C (2 min), and 10 °C/min up to 250 °C (39 min). The total analysis time was 58 min.

**Figure 1:**
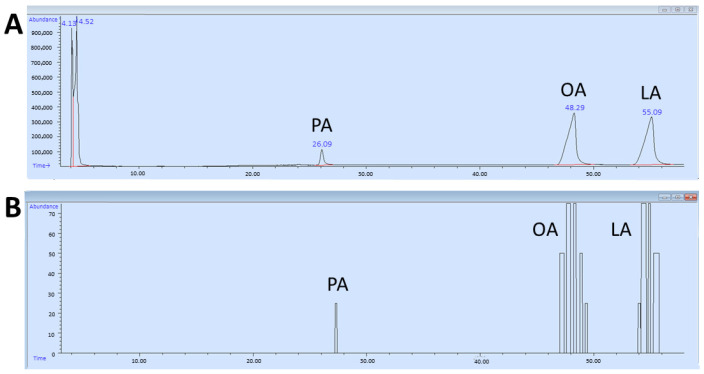
Example of a chromatogram (**A**) and an aromagram (**B**) of palmitic (PA), oleic (OA), and linoleic (LA) fatty acids.

During the GC-O experiments, each time an odor was perceived, the participant had to provide information on the perceived intensity, the quality of the odor (if able to provide it), the duration, and evoked hedonic value [73,74,75], having at their disposal a voice recording system and a keypad digitally connected to the PC (GERSTEL ODP recorder 3 for Windows 7). The keypad has 4 buttons that represent an intensity scale. Based on the button pressed, a rating of the perceived odor intensity is recorded: 1 = weak, 2 = distinct, 3 = intense, and 4 = very intense. While the button was pressed, the participant could speak into a microphone and provide a subjective evaluation of the perceived odor. The PC automatically recorded the retention time and the subjective rating of the perceived odor. This resulted in an aromagram (participant’s evaluation) perfectly aligned with the chromatogram (Figure 1). The samples were presented blindly to avoid participant bias during the experiment.

### 2.4. Determination of the Olfactory Threshold for Fatty Acids

For each of the fatty acids, the following 7 solutions were prepared: palmitic acid (PA) 0.75, 1.5, 3, 4.5, 6, 9, and 12 mM; oleic acid (OA) 6, 12, 24, 48, 95, 190, and 380 mM; and linoleic acid (LA) 0.75, 1.5, 3, 6, 12, 24, and 48 mM. The concentrations of oleic acid were in accordance with those used in the study by Kindleysides et al. [76]. The concentrations of palmitic acid were dictated by the solubility problems of this molecule, while those of linoleic acid were chosen on the basis of preliminary experiments. To neutralize the differences in concentration due to different solubility, the 7 concentrations were marked as dilution steps with a decreasing number from 1 (for the highest concentration) to 7 (for the lowest concentration), like the Sniffin’ Sticks Threshold test. A known amount of fatty acid (20 µL) was dispensed onto a strip of absorbent paper (1 × 6 cm) just before starting the experiment. The fatty acid threshold test was performed following the same protocol as the standardized Thre-test of the Sniffin’ Sticks test. The experimenter had seven triplets of blotting paper strips, each consisting of one paper containing the fatty acid solution and two papers filled with an equal amount of liquid paraffin oil. The participant’s goal was to identify the paper containing the fatty acid (target paper). The test started at the lowest concentration and increased until the participant identified the target paper twice in a row. This was the starting point and represented the first reversal. The test then decreased by a triplet until the participant made an error, at which point the triplet increased again (second reversal), and so on for seven reversals; the olfactory threshold is defined as the average of the dilution steps of the last four reversals. Each triplet was presented at an interval of approximately 20 s. The score assigned to each participant ranged between 1 and 7. Participants who scored higher showed a lower olfactory threshold and vice versa.

### 2.5. Statistical Analysis

Fisher’s method was used to evaluate differences in the distribution of subjects based on their ability to perceive the odors of fatty acids during elution from the chromatographic column or not, also separately between females and males.

One-way MANOVA was used to analyze the effect of TDI status and sex on the in-tensity with which individuals perceived the odors of fatty acids (during elution from the gas chromatographic column) and on the olfactory threshold.

Repeated-measures ANOVA was used to verify the presence of differences in the in-tensity with which individuals perceived the odors of fatty acids (during elution from the gas chromatographic column) and in the olfactory threshold, based on TDI olfactory status and sex.

The assumptions of homogeneity of variance and sphericity (where applicable) were verified. If the sphericity assumption was not met, Greenhouse–Geisser or Huynh–Feldt correction was applied to modify the degrees of freedom. If the assumption of homogeneity of variance was not violated, post hoc comparisons were conducted using the Tukey HSD test [77]. *p* values < 0.05 were considered significant. Statistical analysis was performed using STATISTICA for WINDOWS (version 7.0; StatSoft Inc., Tulsa, OK, USA).

Pearson’s correlation test (if the variables had a normal distribution) or Spearman’s correlation test (if the normality assumption was not met) were used to assess the relationship between the intensity with which each participant, also separately for females and males, perceived the odor of fatty acids (as they were eluted from the gas chromatographic column) and their olfactory threshold for each fatty acid. Statistical analyses were performed using GraphPad Prism 8.1 software (GraphPad Software, San Diego, CA, USA). The significance level was set at alpha = 0.05; *p* < 0.05 values were considered significant.

## 3. Results

The distribution of subjects according to their ability to perceive the odor of palmitic, oleic, and linoleic fatty acids or not, as eluted from the chromatographic column, is shown in Table 1.

Table 2 shows the distribution of females and males who did or did not perceive the odor of palmitic, oleic, or linoleic fatty acids, as they were eluted from the chromatographic column. The results indicate that there was a different ability to perceive palmitic acid (saturated) and oleic and linoleic fatty acids (unsaturated) in the case of females (χ^2^ = 12.88, *p* = 0.0016; Fisher method), but not in the case of males (χ^2^ = 2.67, *p* = 0.26; Fisher method).

**Figure 2:**
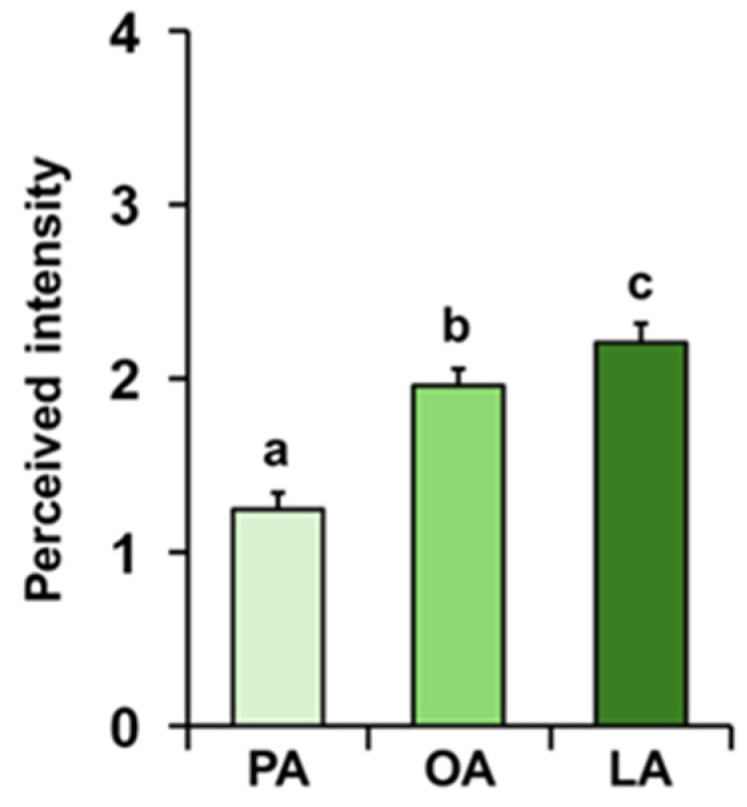
**Perceived intensity of fatty acids during GC-O experiments.** Mean value ± SE of the intensity with which each individual perceived the odors of palmitic (PA), oleic (OA), and linoleic (LA) acids during the GC-O experiments. Different letters indicate significant differences in perceived intensity for the different fatty acids (*p* < 0.05; Tukey HSD test following repeated-measures ANOVA).

The mean values ± SE of the intensity with which the odors of palmitic (PA), oleic (OA), and linoleic (LA) acids were perceived by each individual during the GC-O experiments are shown in Figure 2. Repeated-measures ANOVA revealed the presence of a significant increasing order in the perceived intensity (F _2, 138_ = 46.264; *p* < 0.0001). In particular, post hoc analyses showed that PA was perceived with the lowest intensity, followed by OA with intermediate intensity, and finally LA with the highest intensity (PA-OA: *p* < 0.0001; OA-LA: *p* < 0.05; Tukey HSD test).

**Figure 3:**
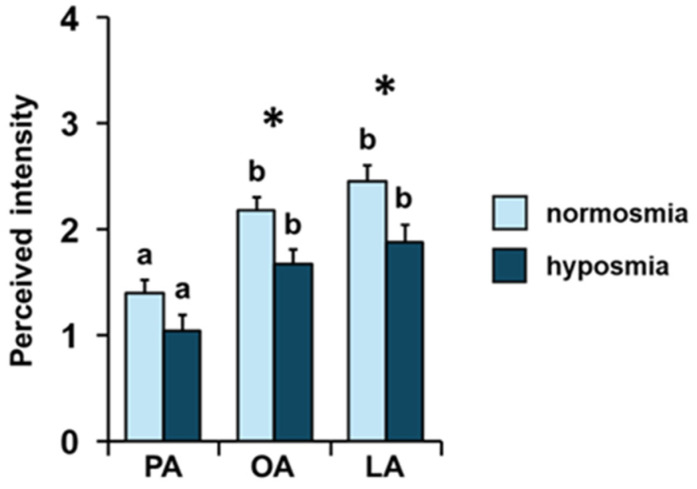
**Effect of the TDI olfactory status on perceived intensity of fatty acids.** Mean value ± SE of the intensity with which each individual perceived the odors of palmitic (PA), oleic (OA), and linoleic (LA) acids during GC-O experiments, according to their TDI status. * indicates significant differences between individuals with normosmia or hyposmia for the same FA (*p* < 0.02; Tukey HSD test following one-way MANOVA). Different letters indicate significant differences in perceived intensity for the different fatty acids within the same olfactory status (*p* < 0.001; Tukey HSD test following repeated-measures ANOVA).

Repeated-measures ANOVA showed that, both among normosmic and hyposmic individuals for their TDI olfactory status, there was an increasing order of perceived intensity for the three fatty acids considered (F _2, 136_ = 0.60; *p =* 0.55; Figure 3). For both olfactory conditions, subsequent post hoc tests showed the following order of intensity: palmitic < oleic = linoleic (normosmia: PA-OA *p* < 0.001, PA-LA *p* < 0.001, and OA-LA *p =* 0.37; hyposmia: PA-OA *p* < 0.005, PA-LA *p* < 0.001, and OA-LA *p =* 0.78; Tukey HSD test). Furthermore, one-way MANOVA revealed the significant effect of TDI olfactory status on perceived intensity (F _3, 66_ = 2.95; *p =* 0.039). Normosmic individuals perceived the odors of OA (*p* = 0.008; Tukey HSD test) and LA (*p =* 0.013; Tukey HSD test) with a higher intensity than hyposmic individuals, while no difference was found in relation to TDI olfactory status for the odor of PA (*p =* 0.085; Tukey HSD test).

**Figure 4:**
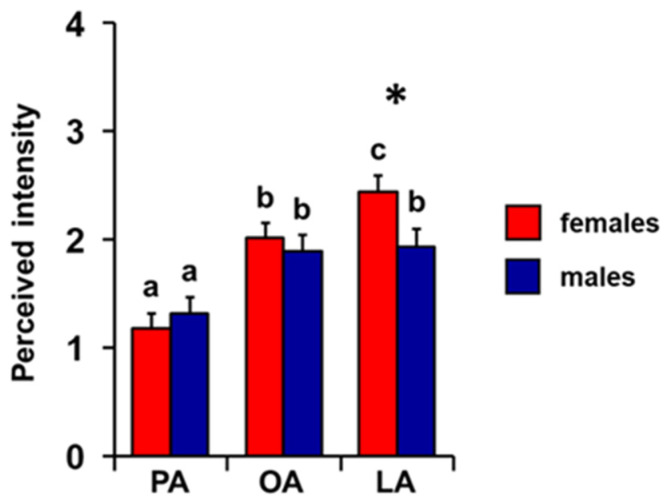
**Effect of sex on perceived intensity of fatty acids.** Mean value ± SE of the intensity with which each individual perceived the odors of palmitic (PA), oleic (OA), and linoleic (LA) acids during the GC-O experiments, separately in females and males. * indicates significant differences between females and males for the same FA (*p =* 0.029; Tukey HSD test following one-way MANOVA). Different letters indicate significant differences in perceived intensity for the different fatty acids, within the same sex (*p =* 0.03; Tukey HSD test following repeated-measures ANOVA).

Repeated-measures ANOVA showed that there is an increasing order of perceived intensity for the three fatty acids considered even when individuals are considered separately as females and males (F _2, 136_ = 5.15; *p =* 0.007; Figure 4). In particular, subsequent post hoc tests showed the following increasing order of perceived intensity: PA < OA < LA and PA < OA = LA, for females and males, respectively (females: PA-OA *p* < 0.001, OA-LA *p =* 0.05; males: PA-OA *p* < 0.005, PA-LA *p* < 0.001, and OA-LA *p =* 0.79; Tukey HSD test). Furthermore, one-way MANOVA revealed the significant effect of sex on perceived intensity (F _3, 66_ = 3.31; *p =* 0.026). In addition, females showed a greater perception for the LA odor of LA (*p =* 0.029; Tukey HSD test), while no sex-related difference was found for the PA or OA odors (*p* > 0.05; Tukey HSD test).

**Figure 5:**
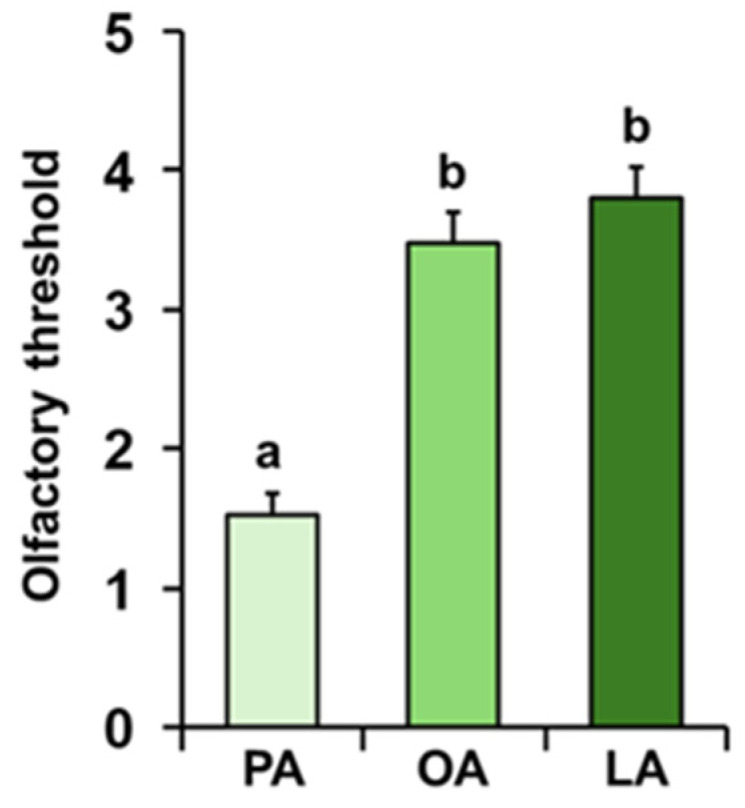
**Olfactory threshold of fatty acids.** Mean value ± SE of the olfactory threshold of individuals for the odors of palmitic (PA), oleic (OA), and linoleic (LA) acids. Different letters indicate significant differences for the different fatty acids (*p* < 0.001; Tukey HSD test following repeated-measures ANOVA).

The mean values ± SE of the olfactory threshold presented by each individual for the odors of palmitic, oleic, and linoleic acids are shown in Figure 5. Repeated-measures ANOVA revealed the presence of a significant decreasing order of threshold (F _2, 138_ = 49.152; *p* < 0.0001). In particular, post hoc analyses showed that PA presents a lower threshold score than OA and LA odors, according to the following order: PA < OA = LA (PA-OA: *p* < 0.001; PA-LA: *p* < 0.001; and OA-LA: *p* > 0.05; Tukey HSD test).

**Figure 6:**
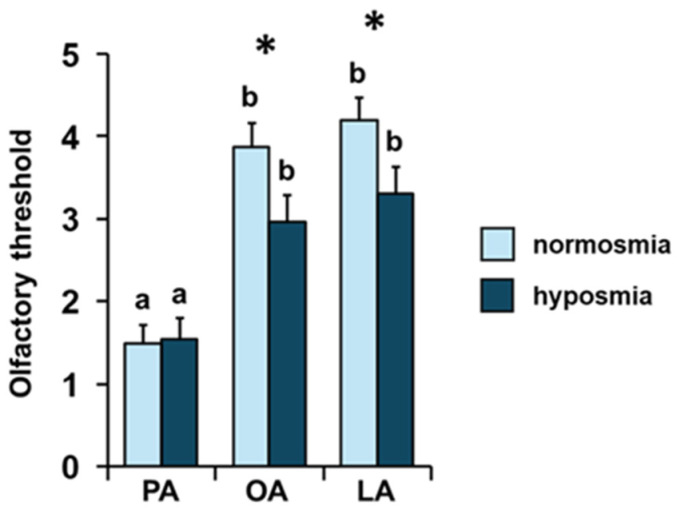
**Effect of the TDI olfactory status on the olfactory threshold of fatty acids.** Mean value ± SE of the olfactory threshold of individuals for the odors of palmitic (PA), oleic (OA), and linoleic (LA) acids, according to their TDI status. * indicates significant differences between individuals with normosmia or hyposmia for the same fatty acid (*p* < 0.05; Tukey HSD test following one-way MANOVA). Different letters indicate significant differences in the olfactory threshold for the different fatty acids within the same olfactory status (*p* < 0.005; Tukey HSD test following repeated-measures ANOVA).

The mean values ± SE of the olfactory threshold score for the odors of PA, OA, and LA obtained by the individuals according to their TDI olfactory status are shown in Figure 6. Post hoc analyses subsequent to one-way MANOVA (F _3, 66_ = 2.08; *p* = 0.11) showed that normosmic individuals reached a higher olfactory threshold score than hyposmic ones for the odors of OA (*p* < 0.05; Tukey HSD test) and LA (*p* < 0.05; Tukey HSD test), while no difference was found for PA between normosmic and hyposmic individuals (*p* > 0.05; Tukey HSD test). Furthermore, post hoc tests following repeated-measures ANOVA (F _2, 136_ = 2.42; *p* = 0.093) showed, for both normosmic and hyposmic individuals, the following increasing order of the olfactory threshold score: PA < OA = LA (*p* < 0.005; Tukey HSD test).

**Figure 7:**
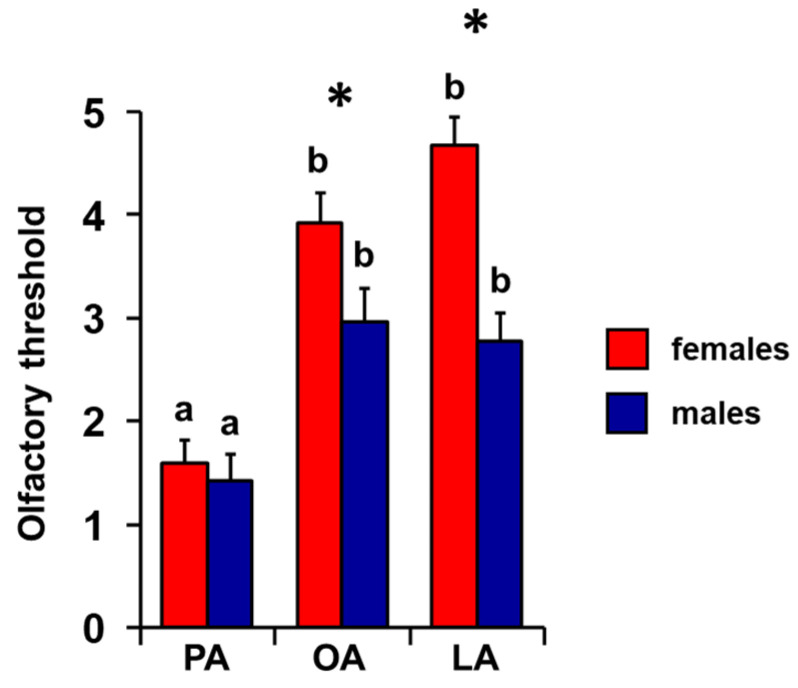
**Effect of sex on the olfactory threshold of fatty acids.** Mean value ± SE of the olfactory threshold of individuals for the odors of palmitic (PA), oleic (OA), and linoleic (LA) acids, according to sex. * indicates significant differences between females and males for the same FA (*p* < 0.05; Tukey HSD test following one-way MANOVA). Different letters indicate significant differences in the olfactory threshold for the different fatty acids within the same sex (females: *p* < 0.001; males: *p* < 0.005; Tukey HSD test following repeated-measures ANOVA).

The mean values ± SEM of the olfactory threshold score for the odors of PA, OA, and LA obtained by both female and male individuals are shown in Figure 7. One-way MANOVA revealed the significant effect of sex on the olfactory threshold (F _3, 66_ = 8.06; *p* = 0.0001). Post hoc analyses showed that females presented a higher olfactory threshold score than males for the odors of OA (*p* < 0.05; Tukey HSD test) and LA (*p* < 0.001; Tukey HSD test), while no difference was found for PA between females and males (*p* > 0.05; Tukey HSD test). Repeated-measures ANOVA revealed the presence of a significant effect of sex on the olfactory threshold (F _2, 136_ = 6.63; *p* = 0.002). Post hoc analyses showed that, for both sexes, there is a significant increasing order of the olfactory threshold score: PA < OA > LA for females (PA-OA *p* < 0.001; OA-LA *p* < 0.02; Tukey HSD test) and PA < OA = LA for males (PA-OA *p* < 0.001; PA-LA *p* < 0.005; and OA-LA *p* = 0.59; Tukey HSD test).

**Figure 8:**
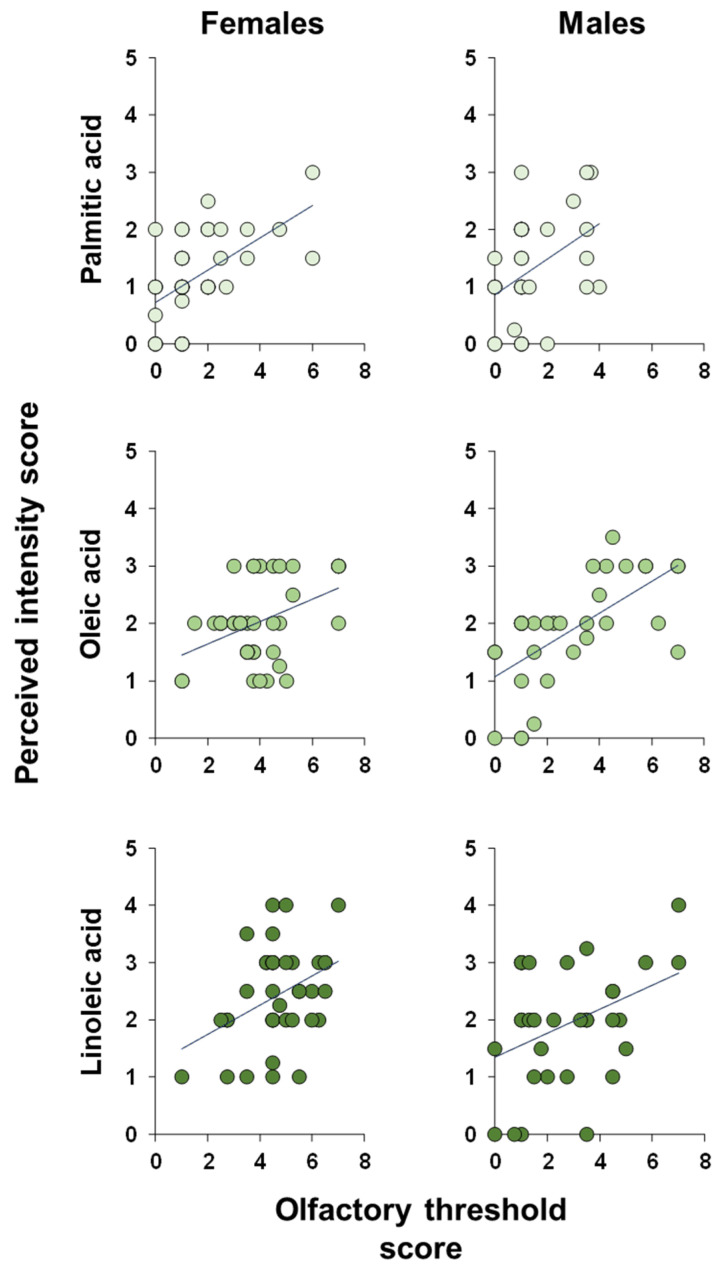
**Correlation analysis.** Correlation analysis between the intensity with which each individual perceived the odors of palmitic, oleic, and linoleic fatty acids while they were eluted from the chromatographic column and his/her olfactory threshold score for the odor of the same fatty acids.

Pearson’s correlation test or Spearman’s correlation test was used to test the correlation between the olfactory threshold and the intensity with which the odors of PA, OA, and LA were perceived during the GC-O experiments. The results shown in Figure 8 indicate a significant positive correlation between the perceived intensity during the GC-O experiments and the olfactory threshold score obtained by each participant for each of the fatty acids considered, both when the individuals are considered as a whole (PA: Spearman’s r = 0.48, *p* < 0.0001; OA: Pearson’s r = 0.56, *p* < 0.0001; and LA: Spearman’s r = 0.63, *p* < 0.0001) and separately in females (PA: Spearman’s r = 0.55, *p* = 0.0004; OA: Pearson’s r = 0.41, *p* = 0.0106; and LA: Pearson’s r = 0.39, *p* = 0.0167) and males (PA: Spearman’s r = 0.40, *p* = 0.023; OA: Pearson’s r = 0.65, *p* < 0.0001; and LA: Pearson’s r = 0.39, *p* = 0.0276).

## 4. Discussion

One of the most debated topics in the literature regarding the perception of the odor of fatty acids is its olfactory pathway and whether the odor is that of the free fatty acid molecule or its oxidation products. Most fatty acids in foods are found in the form of triglycerides and, at room temperature, they rapidly undergo oxidation processes leading to the formation of volatile compounds such as alcohols, aldehydes, and ketones [11,56,78]. However, low concentrations of free fatty acids are also present in some foods [2,3]. The first aim of our study was to evaluate the ability to perceive the odor of fatty acids and measure the olfactory threshold through the orthonasal pathway. The results obtained with the GC-O experiments show that individuals are able to perceive the odors of palmitic, oleic, and linoleic acids as they are eluted from the chromatographic column, even if the number of individuals perceiving the odor of OA and LA is significantly higher than those perceiving the odor of PA. The different ability shown by individuals in the perception of fatty acids has an important functional implication: PA, for which individual perception is lower, has been associated with hypercholesterolemia, inflammatory processes, insulin resistance, high cardiovascular risk and diabetes; OA has been associated with a reduction in blood cholesterol content, oxidative stress, inflammatory state, and blood pressure; and LA has been seen to reduce blood cholesterol levels, particularly LDL, promote the formation of bile acids, increase insulin sensitivity, and reduce blood pressure and the incidence of cardiovascular diseases [5]. Smelling the odor of PA from a distance could allow these individuals to avoid foods high in this fatty acid and therefore, represents a defense mechanism against health risk factors. The greater ability of individuals to perceive the odors of OA and LA would allow them to identify and choose foods that contain these fatty acids, with beneficial effects on health, especially regarding LA, a polyunsaturated fatty acid defined as essential that cannot be synthesized de novo by the body and must be introduced through diet [5]. From this perspective, since this study was limited to examining the olfactory sensitivities of individuals to only a few fatty acids present in foods and consumed with the diet, albeit belonging to different categories (saturated, monounsaturated, and polyunsaturated), it would be interesting to study olfactory sensitivities to other fatty acids of both dietary and functional interest, and their relationship with eating behavior. This would allow us to better understand how perception varies between individuals and in relation to the properties of the molecule, but also how sense of smell can help select foods containing molecules of interest for an individual’s well-being.

Our data also show that the perceived intensity was inversely proportional to the number of double bonds present in the molecule, highest for LA, intermediate for OA, and lowest for PA, and that the olfactory threshold was lowest for LA, intermediate for OA, and highest for PA. These results, taken together, highlight that both the ability to perceive odors and the intensity with which fatty acids are perceived are inversely proportional to their lipophilicity. As the number of double bonds increases, the water solubility of the molecules increases, and this facilitates the fatty acid to reach the dendritic terminal and bind to its olfactory receptor [79]. Remembering that the ability to perceive and intensity of the perception of an odor depend on the activation of the olfactory sensory neurons, a greater number of receptor–odor bonds determines a more intense depolarization; therefore, a greater number of action potentials will reach the central nervous system and the perception of the odor will be more intense. This is of particular importance if we consider that the olfactory receptors are immersed in the perireceptor space, a thin layer of mucus rich in water and glycoproteins [80,81,82,83,84,85]. We can hypothesize that oleic and linoleic fatty acids, which are more water-soluble due to their double bonds, are able to cross the mucus layer more easily and therefore reach the olfactory receptors. We can also assume that oleic and linoleic fatty acids are able to bind more easily to odor-binding proteins (OBPs), reaching the olfactory receptors more easily. In this regard, future studies will be directed to study the polymorphism of the gene-encoding OBPs, whose expression and function have been associated with variations in olfactory function in both healthy and pathological individuals, in their ability to perceive single molecules and the intensity with which they are perceived [66,73,86,87,88,89].

In agreement, correlation analyses show that the intensity with which fatty acids are perceived is directly proportional to their olfactory threshold: the higher the score obtained during the olfactory threshold test, the lower the olfactory threshold and the greater the intensity with which the odors of palmitic, oleic, and linoleic acids are perceived. These results are consistent with and confirm previous studies demonstrating that the ability to perceive the molecules that compose the complex aromas of foods, such as coffee and banana, and the intensity with which these molecules are perceived, are directly correlated with the olfactory functions of individuals [42,72].

The ability to perceive the odor of fats via the orthonasal pathway, and therefore at a distance, is seen as an advantageous adaptation, as it would allow us to identify the caloric content of food even before ingesting it, thus facilitating the recognition and choice of food sources rich in calories, which can be easily accumulated over long periods of time [30]. On the basis of these considerations, the second aim of our study was to evaluate the differences in the perceptions of fatty acids in relation to the olfactory functions of individuals. The results we obtained highlight that, compared to individuals with hyposmia, normosmic individuals show a higher sensitivity and a lower olfactory threshold for OA and LA. This aspect is very important considering that the decline of the sense of smell leads individuals to choose less healthy foods [24,25]. In detail, people with a reduced sense of smell have been shown to prefer foods rich in sugars and fats, enriched with salt and spices, and present a low adherence to the Mediterranean diet, which is rich in fruit and vegetables and has a low intake of fiber and cereals [23,25,27,29]. Individuals with hyposmia have a higher threshold for palmitic acid, an important saturated energy source and a precursor of other fatty acids, and a lower threshold for oleic and linoleic acids, unsaturated and polyunsaturated, which modulate the permeability of plasma membranes, are precursors of prostaglandins, and have a hypocholesterolemic action [4,5,6,7,8,9,10]. Evolution may have favored the orthonasal perception of fatty acids both because they represent an important energy source and because of the beneficial effects presented by fatty acids such as OA and LA. In this regard, individuals with a higher sensitivity may benefit more by choosing healthier foods than individuals with hyposmia.

Previous studies have shown that females outperform males in both their general olfactory function and their ability to perceive single molecules that compose a complex mixture [42,67], so the final aim of the present study was to evaluate sex-related differences in fatty acid odor perception. Our results show that females perceive LA odor more intensely during GC-O experiments and have a lower olfactory threshold for OA and LA than males. Females, therefore, also show better olfactory performance than males in the perception of molecules of food interest and with health benefits, such as unsaturated and polyunsaturated fatty acids. We hypothesize that this ability may allow females to identify foods with a nutritional content beneficial to health more easily than males: this could partly explain the greater adherence of females to the Mediterranean diet [23]. This diet, characterized by a high consumption of plant-based foods, such as olive oil rich in OA, and blue fish, rich in polyunsaturated fatty acids, such as LA, is known for its protective effects against cardiovascular and neurodegenerative diseases, diabetes, cancer, and obesity [90,91,92,93]. Given the contribution of the polymorphism of the gene encoding the Kv1.3 channels to interindividual variability and sex-related differences in olfactory function [51,52,94], these findings suggest studying its role in fatty acid perception in the future.

## 5. Conclusions

In conclusion, the results of this study confirm the orthonasal perception of fatty acids and show that it is directly related to the number of double bonds present in the molecule and inversely related to its lipophilicity. Furthermore, a direct relationship has been highlighted between the olfactory threshold and perceived intensity: a lower threshold corresponds to a higher perception. These aspects seem to be related to the olfactory status of individuals and their sex: normosmic individuals and females show a lower olfactory threshold and a higher perception intensity than individuals with a reduced sense of smell and males, respectively.

## Figures and Tables

**Table 1:** Distribution of subjects who did or did not perceive the odor of palmitic, oleic, and linoleic fatty acids.

Variable	YES	NO	*p*-Value
	*n* (%)	*n* (%)	
Palmitic acid	55 (78.57)	15 (21.43)	0.001
Oleic acid	67 (95.71)	3 (4.29)	
Linoleic acid	66 (94.29)	4 (5.71)	

*p*-value derived by means of Fisher’s test. N = 70.

**Table 2:** Distribution of females and males who did or did not perceive the odor of palmitic, oleic, and linoleic fatty acids.

	Group	YES	NO	*p*-Value
	Variable	*n* (%)	*n* (%)	
Females	Palmitic acid	29 (76.32)	9 (23.68)	0.002
	Oleic acid	37 (97.37)	1 (2.63)	
	Linoleic acid	37 (97.37)	1 (2.63)	
Males	Palmitic acid	26 (81.25)	6 (18.75)	0.263
	Oleic acid	30 (93.75)	2 (6.25)	
	Linoleic acid	29 (90.63)	3 (9.37)	

*p*-value derived by means of Fisher’s test. N = 38 females; N = 32 males.

## Data Availability

The data presented in this study are available on request from the corresponding author. The data are not publicly available due to restrictions (e.g., privacy or ethical).
